# Detection and Classification of Finer-Grained Human Activities Based on Stepped-Frequency Continuous-Wave Through-Wall Radar

**DOI:** 10.3390/s16060885

**Published:** 2016-06-15

**Authors:** Fugui Qi, Fulai Liang, Hao Lv, Chuantao Li, Fuming Chen, Jianqi Wang

**Affiliations:** Department of Medical Electronics, School of Biomedical Engineering, Fourth Military Medical University, Xi’an 710032, China; qifgbme@outlook.com (F.Q.); liangfulai@fmmu.edu.cn (F.L.); fmmulvhao@fmmu.edu.cn (H.L.); chuantaoli@hotmail.com (C.L.); fumingfmmu@outlook.com (F.C.)

**Keywords:** finer-grained human activity, comprehensive range accumulation, human micro-Doppler, through-wall, support vector machine

## Abstract

The through-wall detection and classification of human activities are critical for anti-terrorism, security, and disaster rescue operations. An effective through-wall detection and classification technology is proposed for finer-grained human activities such as piaffe, picking up an object, waving, jumping, standing with random micro-shakes, and breathing while sitting. A stepped-frequency continuous wave (SFCW) bio-radar sensor is first used to conduct through-wall detection of finer-grained human activities; Then, a comprehensive range accumulation time-frequency transform (CRATFR) based on inverse weight coefficients is proposed, which aims to strengthen the micro-Doppler features of finer activity signals. Finally, in combination with the effective eigenvalues extracted from the CRATFR spectrum, an optimal self-adaption support vector machine (OS-SVM) based on prior human position information is introduced to classify different finer-grained activities. At a fixed position (3 m) behind a wall, the classification accuracies of six activities performed by eight individuals were 98.78% and 93.23%, respectively, for the two scenarios defined in this paper. In the position-changing experiment, an average classification accuracy of 86.67% was obtained for five finer-grained activities (excluding breathing) of eight individuals within 6 m behind the wall for the most practical scenario, a significant improvement over the 79% accuracy of the current method.

## 1. Introduction

In recent years, non-contact penetrating detection and classification technology directed toward human activity have attracted broad research attention due to its promising and crucial value in practical applications such as public security and protection, anti-terrorism operations, and disaster rescue [1,2,3]. Undoubtedly, if finer-grained human activities (e.g., waving, jumping when staying in roughly one spot, picking up an object, or standing in roughly one spot with random micro-movements) behind a wall can be detected without contact and classified accurately, soldiers or police officers could grasp detailed information about the actions of a target without risking direct confrontation. Furthermore, at disaster sites in which the environment is temporarily hazardous or impossible for the approach of rescuers, such as after a chemical explosion or a seismic landslide, if the remote detection of trapped individuals by penetrating the wall or chemical smoke can be realized and human motion features can be identified, the rescuers will be provided with critical motion state information of the trapped in advance. Therefore, the through-wall detection, analysis and classification of finer-grained human activities would be very helpful in decision-making and formulating rescue strategies, greatly improving rescue efficiency and operational effectiveness.

Bio-radar is a novel kind of radar combining the technology of biomedical engineering and radar, which aims to detect in a non-contact way vital signs through non-metallic obstacles such as clothes and walls [4]. Ultra-wideband (UWB) bio-radar has gradually become a research hotspot of human location and motion detection [5,6] due to its advantages of strong anti-jamming capability, good penetrability, and high-range resolution. A new form of UWB radar, known as stepped-frequency continuous-wave (SFCW) radar, transmits a series of discrete tones in a stepwise manner, covering the radar bandwidth in the time domain to realize the UWB. Therefore, it has several unique advantages over conventional impulse radio-ultra wideband (IR-UWB) radar [7,8], such as a higher dynamic range and mean power for higher range and resolving power, high reliability, stability of the radar signal, and relatively easy implementation. Therefore, this paper develops a SFCW radar to conduct the through-wall detection of different human finer-grained activities.

To date, relying on the effective analysis tool—the Time-Frequency transform—some complex human activities have already been classified under free space conditions, mainly based on the corresponding micro-Doppler features differences caused by limb movement, such as the classification of waving, standing, breathing, and stooping [1], the classification of one-arm swing walking, two-arm swing walking and no-arm swing walking [9] as well as the classification of armed and unarmed personnel [10]. The experimental results show that the foregoing method realizes highly accurate classification in free space. However, penetrating detection is necessary in practical applications such as anti-terrorism actions and disaster rescue. If the through-wall radar echo is greatly attenuated or the objective is far from the radar, the micro-Doppler features of the motion will become very weak and the signal to noise ratio (SNR) will be greatly reduced, which may reduce the recognition of those features and decrease the classification accuracy [11,12,13]. Therefore, making reasonable and effective enhancements of the weak micro-Doppler features of finer-grained human activity radar signals is crucial. Because the useful information modulated by body movements is distributed in different range bins of the UWB radar signal [14], the most effective way to enhance the micro-Doppler signal is to make full use of the radar signals in different range bins. Currently, the common methods provide range averages [15,16] or range accumulations [17] before analyzing in the time and frequency domains, which are often used for the detection of human respiration and heartbeat signals that feature small movement amplitudes, strong regularity, and good stability. However, for finer-grained human activities that feature large amplitude changes, are non-stationary, and weakly periodic, the movement of each scattering center (each limb structure) of the moving body will be roughly distributed in different range bins. Consequently, both of two common methods will be eliminated because they are likely to miss or change the feature information of the motions.

Therefore, considering the UWB radar signal characteristics of human activity, this paper proposes a comprehensive range-accumulation time-frequency transform (CRATFR) method based on an inverse weight coefficient, which strengthens the micro-Doppler features of human activity signals by making full use of the time-frequency information of different scattering center signals in the different range bins of the UWB radar signals.

After the motion detection and features extraction, our intention is to accurately classify different finer-grained human activities at any position. In this paper, a mature support vector machine (SVM) was adopted for activity classification. However, due to the influence of factors such as through-wall attenuation and detection range changes, it is inevitable that micro-Doppler features will undergo attenuation in any time-frequency distribution. As a consequence, one activity at a given range may have similar eigenvalues derived from micro-Doppler spectrogram as another activity at a different range. If an overall SVM is applied, the possibility of misjudging the corresponding activities at different distance positions will be greatly increased. Thus, we are faced with another challenge: how to reduce the possibility of misjudging different finer-grained human activities as a result of distance changes?

To address this issue, this research introduces an optimal self-adapting support vector machine (OS-SVM) based on prior information of the human position to classify the different finer-grained human activities. Since UWB radar is capable of realizing the accurate through-wall positioning [6,18,19] of the moving body, a corresponding SVM group for the classification of motion states at different positions could be established based on the corresponding eigenvalue data of finer-grained human activities. During the prediction, the radar first senses the environment through the wall. As it is a UWB radar, it has very good range resolution, and therefore, it can locate the target in range very accurately. Then, the corresponding optimal SVM classifier that is tailored to that specific range is selected and applied self-adaptively.

Through the above analysis, the paper finally proposes a through-wall detection and classification technology for finer-grained human activity based on SFCW radar, mainly comprising the acquisition of human activity information, the CRATFR, the extraction of the eigenvalues, and pattern recognition based on the OS-SVM. In this paper, six representative finer-grained human activities are classified by the proposed technology, including piaffe (walking on the spot), picking up an object, waving, jumping, standing with random micro-shakes, and breathing while sitting (all of these activities are implemented when human staying in place).

This paper is organized as follows: Section 2 describes the SFCW radar through-wall detection system and data acquisition environment. Section 3 introduces the methods for finer-grained human activity radar signal processing and classification. Section 4 comprises the performance analysis and verification of the through-wall detection and classification technology for finer-grained human activity using specific experiments, including a through-wall fixed-point experiment and a through-wall variable-position experiment. 

## 2. SFCW Radar Through-Wall Detection System for Finer-Grained Human Activities and Experimental Establishment

**Figure 1:**
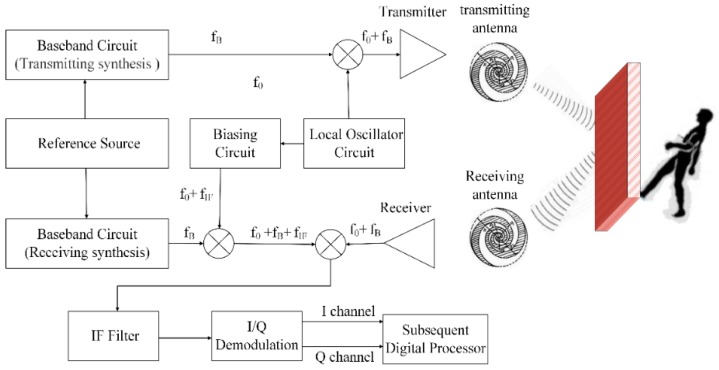
Schematic diagram of the SFCW radar system.

The SFCW radar system is illustrated in Figure 1. The detailed operating parameters are as follows: operation bandwidth, 0.5–3.5 GHz; adjustable number of transmitter step points, 101–301; transmitter frequency sampling interval, 30 MHz; maximum transmitting power, 10 dBm; dynamic range, >72 dB; analog-to-digital conversion (ADC) accuracy, >12 bit; sampling rate, 250 Hz; and maximum unambiguous range, 5 m. The plane logarithmic spiral antenna system consists of a single-transmitting and double-receiving antenna array. Cross polarization is adopted between the transmitting and receiving antennas. During the latter processing, we just use one channel signal derived from one of two receiving antennas which has a better performance.

When detecting human motion, the principles of the SFCW radar may be described as follows: assuming that the pulse repetition interval (PRI) of the SFCW signal is *T*, the transmitting pulse width is *T*, the initial work frequency is *f*_0_, the frequency step increment is Δ*f*, and the stepped frequency is *N*, then the stepped frequency signal *S_t_*(*t*) may be written as follows:

(1) $$S_{t}\left(t\right)=\overset{N-1}{\underset{n=0}{\sum }}rect\left(\frac{t-\left(n+\frac{1}{2}\right)*T}{T}\right)exp\left\{j\left(2\pi \left(f_{0}+n\Delta f\right)t+\varphi_{0}\right)\right\}$$

where, $rect\left(\frac{t}{T}\right)=\{\begin{array}{c} 1\;-\frac{T}{2}\leq t<T/2\\ 0\;\mathrm{else}\; \end{array}$, *n* = 0,1,2,…*N* − 1 indicates the modulation period and $\varphi_{0}$ refers to the initial phase of the transmitted signal.

Assuming that the radial distance of the objective relative to the radar is *R*(*t*), the target echo can be described as follows:

(2) $$S_{r}\left(t\right)=\overset{N-1}{\underset{n=0}{\sum }}rect\left(\frac{t-\left(n+\frac{1}{2}\right)*T-\tau \left(t\right)}{T}\right)\rho_{n}exp\left\{j\left(2\pi \left(f_{0}+n\Delta f\right)\left(t-\tau \left(t\right)\right)+\varphi_{0}\right)\right\}$$

where, *ρ*_n_ refers to the backscattering coefficient of the objective and is assumed constant within the radar observation period, which is set as ρ uniformly;$\;\tau \left(t\right)=\frac{2R\left(t\right)}{c}\;$refers to the time delay of the objective at *R*(*t*) relative to the transmitting moment, and *c* refers to the speed of light. 

Frequency mixing and a low-pass filtering process are conducted on the radar echo, for a sampling interval *T*, so that a sampling point can be obtained within each of the modulation periods. Assuming that the objective’s range remains unchanged within a frame signal duration, the zero intermediate frequency echo of the objective can be discretized as:

(3) $$S\left(m,n\right)=\rho exp\left\{-j2\pi \left(f_{0}+n\Delta f\right)\tau \left(\left(m-1\right)NT+nT\right)\right\}\;=\rho exp\left\{-j2\pi \left(f_{0}+n\Delta f\right)\tau \left(m,n\right)\right\}$$

where *m* = 1,2,3,…*M* indicates the quantity of sampling frames, τ(m,n)=τ((m−1)NT+nT) refers to the sampling of τ(t) at (m−1)NT+nT moment.

The formula above is the common form of the SFCW radar echo. If *m* is a fixed value, the target echo can be regarded as the sampling on different frequencies at the same moment. Then, a one-dimensional time-range image of the objective motion can be obtained through the computation of the inverse discrete Fourier transform (IDFT), so as to conduct Doppler analysis on the time-range image sequence of objective range bins.

**Figure 2:**
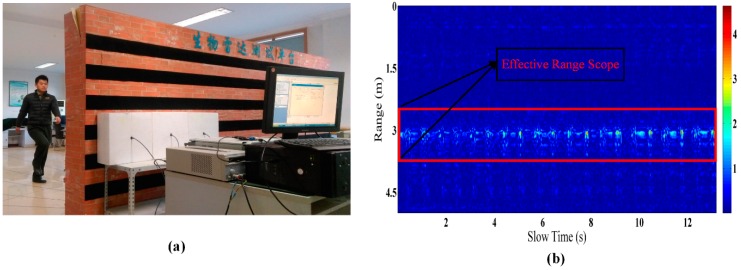
(**a**) Through-wall detection of human activity. (**b**) Preprocessed SFCW radar signal of piaffe-type movement.

A through-wall detection experiment of human activity by the SFCW radar is shown in Figure 2a. The subject is on one side of the laboratory brick wall (which is about 30 cm thick), and faces the radar which is placed against the wall. During each acquisition of data, only one subject stands on one side of the wall. The subject executes specific movements according to personal motion habits and characteristics.

## 3. Analysis and Classification of Finer-Grained Human Activities

If the weak and fuzzy micro-Doppler time-frequency features due to through-wall attenuation can be enhanced effectively, efficient recognition of finer-grained human activity can be certainly realized by extracting reasonable eigenvalues and adopting an appropriate pattern recognition method. This section first introduces the working principles of the CRATFR method based on an inverse weight coefficient, and then proves the enhancement effects. Then, eight eigenvalues are selected which reflect the motion characteristics according to the features of the SFCW radar signal spectrum of the finer-grained human activity, and the extraction method and its significance are briefly described. Finally, this section introduces the working principles of the proposed OS-SVM pattern recognition and classification method based on prior human position information.

### 3.1. Principles of Comprehensive Range Accumulation Time-Frequency Transform (CRATFR) Based on Inverse Weight Coefficient

During the detection of human activity using the UWB radar, different delay echoes obtained from objectives at different distances from the UWB radar can be acquired at the same time. These echoes are stored in a two-dimensional data matrix ***R*** after being amplified and sampled:

(4) $$R=\left\{r^{\left(m\right)}\left[n\right]:m=1,\ldots ,M,n=1,\ldots N\right\}$$

where *m* and *n* indicate the range and time indexes, respectively. *M* refers to the quantity of sampling points along the distance axis, and determines the radar detection distance. *N* refers to the quantity of sampling points along the time axis, which determines the total time of the data together with the sampling frequency and can be regarded as a series of signals on the range axis: $\text{sig}=\left\{sig_{i}|\left(t\right):i=1,..N\right\}$. 

As shown in Figure 2b, a preprocessed SFCW radar signal of piaffe activity is obtained after conducting successive preprocessing steps such as background removal and low-pass filtering (cutoff frequency, 60 Hz) on the original echo. Obviously, the motion features are mainly distributed in the range scope of 2.5–3.8 m, referred to as the effective range scope. The motion changes can be clearly seen in the preprocessed echo image, and each scattering center of the human body will span several range bins during movement. As Smith *et al.* indicated, when using a time-frequency transform to extracting the micro-Doppler features for motion feature analysis [20], the use of different range bin signals is very important.

**Figure 3:**
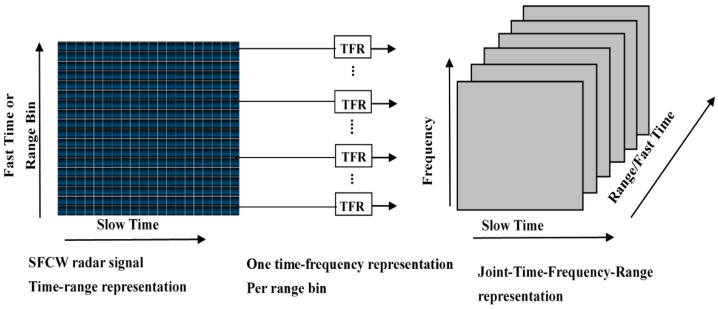
The joint time-frequency-range representation of the UWB radar signal.

To verify the advantages of the proposed CRATFR method based on inverse weight coefficients, this paper takes the conventional method (time-frequency transform (TFR), based on range average) as the reference method. The basic principles of the reference and proposed methods are as follows:

1. TFR based on range average method. In this approach, the averaging of different range bin signals extracted within an effective range scope with an interval is first conducted to obtain *sig_average_*, and then, a time-frequency analysis is performed:
  (5) $$\;sig_{average}=\overset{k}{\underset{1}{\sum }}\frac{sig_{i*N}}{k}\;i=1\ldots \ldots .k$$
  where *N* indicates the length of the interval, and *k* indicates the total number of distance bin signals.
2. CRATFR based on inverse weight coefficient. In this case, the joint time-frequency-range representation (JTFRR) [21] is first obtained, as shown in Figure 3.

Then, the time-frequency transform of each range bin signal is performed to obtain the time-frequency representation (TFR). The TFRs of different range bins are collected in order to obtain the JTFRR three-dimensional spectrum of the complete human-activity UWB radar signal. As shown in Figure 3, the three coordinate axes indicate the range, time, and frequency, respectively. Then, the accumulation of the TFRs obtained from each range bin signal according to the corresponding inverse weight coefficient along the range axis is taken. Finally, the CRATFR of the whole motion signal is obtained.

To enhance the micro-Doppler features formed by the motion of limbs, this paper adopted an inverse weight coefficient to conduct the comprehensive range accumulation of different time-frequency spectra, shown in Equation (6):

(6) $$s_{d}=\omega_{1}*s_{1}+\omega_{2}*s_{2}+....\omega_{n}*s_{n}$$

where *ω_ι_* indicates the weight corresponding to the TFR of range bin *i*, while *s_i_* indicates the corresponding TFR. For *i* = 1,2,… *n*, *n* indicates the number of range bins within the effective range scope. 

The selection of the weight coefficient ω is based on the energy characteristics of the SFCW radar finer-grained human activity signal. Taking the piaffe signal shown in Figure 2b as an example, within the effective range of motion features, the middle part of the range axis mainly comes from torso movement, with its large scattering area, and features strong energy. The energy gradually weakens as the range moves away from the middle, because the signal arises mainly from limb movements, with their small scattering areas (*i.e.*, arms and legs). When the signal through the wall is attenuated or the objective moves away from the radar, the micro-Doppler features caused by arms and legs will attenuate sharply. Hence, we apply a larger weight to the range bin TFR with weaker energy, while a smaller weight is given to the range bin TFR with stronger energy. Based on this method, the micro-Doppler features afforded by the movement of limbs will be enhanced effectively in the time-frequency domain. 

**Figure 4:**
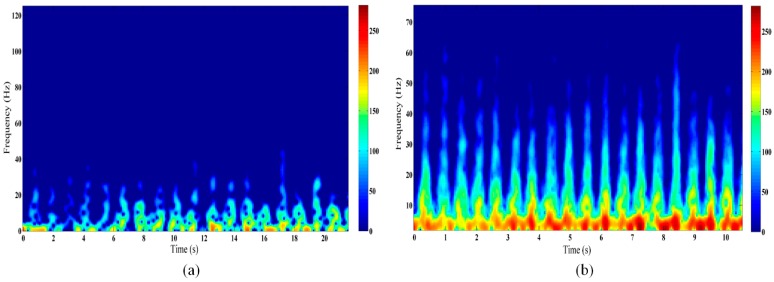
Time-frequency spectra of SFCW radar signal for piaffe at a distance of 4 m behind the wall, based on different range accumulation methods: (**a**) Reference method and (**b**) Proposed method.

In this paper, a short-time Fourier transform (STFT) (0.42 s Hanning window) is applied to generate the corresponding TFR for each range bin signal. Because we have conducted the operation of taking absolute value on the output channels before reducing the background based on amplitude, only the positive frequency can be showed in the Doppler spectra. Comprehensive time-frequency analyses based on the reference and proposed methods were conducted for the piaffe activity at a distance of 4 m behind the wall, with the results shown in Figure 4. As can be seen from Figure 4, more distinguishable, regular, and stronger motion features can be extracted from the high frequency positions by the proposed method than the reference, due to its advantages of the micro-Doppler enhancement effect and its excellent immunity to interference by noise and clutter. Furthermore, further experiments indicated that the advantages of this method are highlighted during long-range through-wall detection.

**Figure 5:**
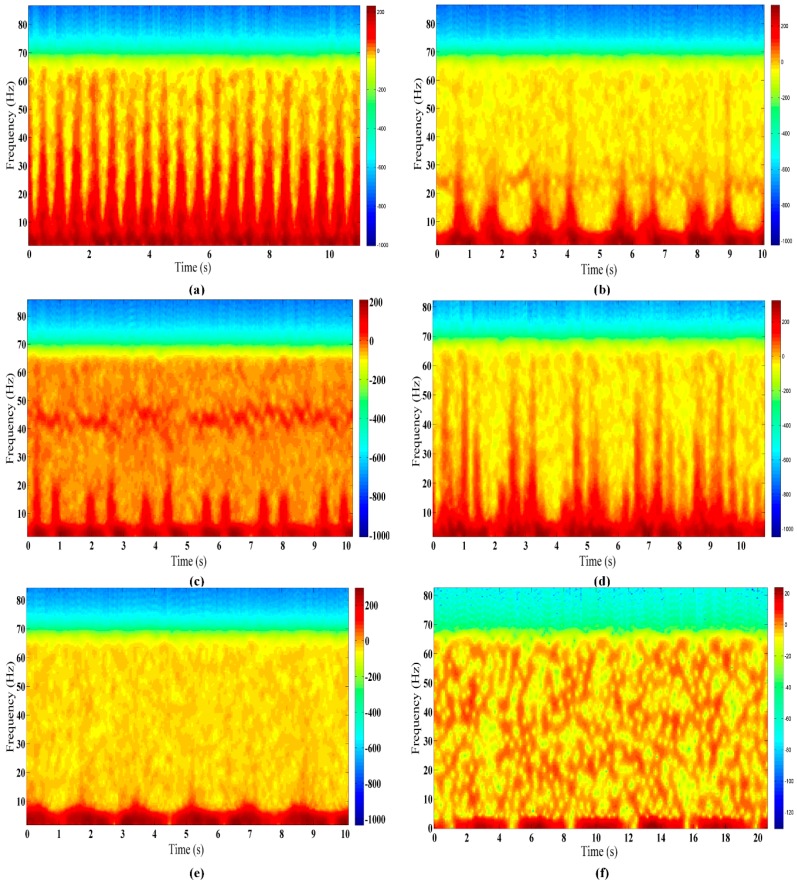
CRATFR spectrograms of six finer-grained human activities performed at 3 m behind a wall: (**a**) piaffe; (**b**) picking up an object; (**c**) waving; (**d**) jumping; (**e**) standing with random micro-shakes; and (**f**) breathing while sitting.

Finally, the CRATFR spectrograms of six different finer-grained human activities from one particular subject are shown in Figure 5. 

The CRATFR spectrogram based on the inverse weight coefficient is capable of clearly distinguishing the rhythmic changes due to different activities, and the micro-Doppler features formed by trunk movement are obviously different from those formed by limb movements. The specific performance can be divided into the following categories:

1. The human body stays roughly in place during finer-grained human activities. Since the scattering area of the human trunk is the largest, the strongest part of the radar echo spectrogram comes from the low-frequency Doppler signals formed by torso motion. At the same time, the periodic micro-Doppler signals above the torso arise mainly from the legs and arms.
2. Except for breathing while sitting, the Doppler frequencies of the other five activities basically range from 0 to 7 Hz.
3. In terms of the micro-Doppler characteristics, the highest frequencies from piaffe and jumping can reach more than 60 Hz, but piaffe has more regular micro-Doppler features. The spectral features due to picking up an object are generally similar to those of waving, with the highest frequency around 25 Hz, while its micro-Doppler features occupy a larger spectral area than those of waving, since waving only involves the movement of a single arm within a small range, in addition to body movement. Compared with the other four activities, those of standing with random micro-shakes and breathing while sitting exhibit fewer micro-Doppler features. However, the former activity has stronger Doppler features accompanied by discontinuous weak Doppler features formed by the random shaking of the arms.
4. The activity periods of all six activities differ significantly.

### 3.2. Feature Extraction

**Figure 6:**
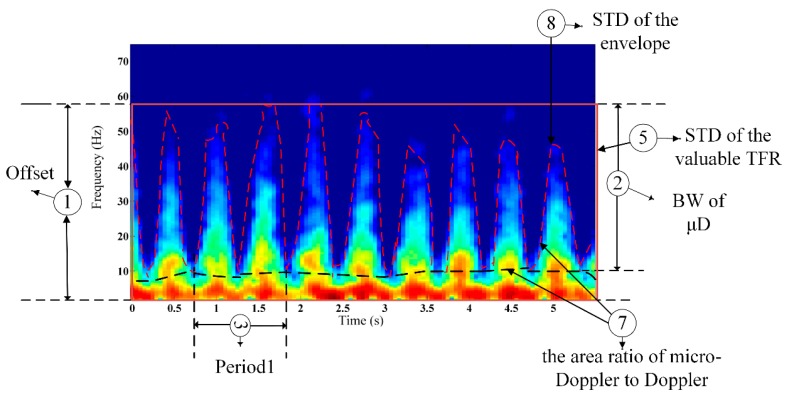
Some of the eight features extracted from the CRATFR spectrogram.

According to the CRATFR spectrograms shown in Figure 5, different activities reveal interesting and distinct features. To facilitate the extraction of distinct characteristics from the spectra of different activities, noise submerged in the Doppler signal is removed according to threshold values obtained by conducting contrastive analysis on background signals and the measured signals of human activity. As shown in Figure 6, the rest of the spectrogram will substantially comprise only activity information.

Based on the analysis of the spectra of the different activities above, eight features are selected to characterize the Doppler signatures; Some of these are illustrated in Figure 6: (1) the offset of the Doppler signal; (2) the band-width (BW) of the micro-Doppler signal; (3) the main period of human body activity; (4) the secondary period of human body activity; (5) the standard deviation (STD) of the valuable portion of the CRATFR (non-zero row); (6) the ratio of the total BW of the Doppler signal to the period of limb activity; (7) the area ratio of the limb characteristics to the torso characteristics in the total valuable portion of the CRATFR (non-zero value); and (8) the STD of the envelope (upper envelope) of the CRATFR.

Characteristic (1) is related to the speed of limb motion, and faster swings of the arms or legs will cause a larger BW. Factor (2) describes the frequency range formed by limb movement, namely, the speed variation range, while (3) describes the time required for completing a motion. Feature (4) describes the cycle formed by minor limb movements except the overall motion cycle during the motion process, and (5) is related to the dynamic range of the activity. Characteristic (6) shows the spectral distribution features of the whole motion, which may be large if the activity has a short cycle and intense motion, such as piaffe, or may be small, as when picking up an object. (7) is related to the scattering areas and amplitudes of the limb and trunk movements involved in the motion, and (8) reveals the non-stabilization of the motion.

### 3.3. Classification by Optimal Self-Adaption Support Vector Machine (OS-SVM) Based on Prior Human Position Information

Having described the extraction of features, we next turn to classification by the SVM in combination with eigenvectors. As a classifier [22,23] for solving nonlinear boundary problems, SVM is mainly used for optimizing and generating corresponding models based on a training-data support feature and corresponding known category name. Based on this model, the testing data can be classified accurately through the support vector. Thus, we adopted the sophisticated LibSVM3.1 algorithm developed by Chang and Lin [24], which can realize automatic multi-classification. During parameter selection, a radial basis function was selected in advance as the kernel function of the SVM on the basis of experimental results and the characteristics of this kernel function [25]. For two other parameters, the penalty parameter *c* and the kernel parameter *g*, we utilized four-fold cross-validation to conduct automatic selection based on the minimum classification error principle.

**Figure 7:**
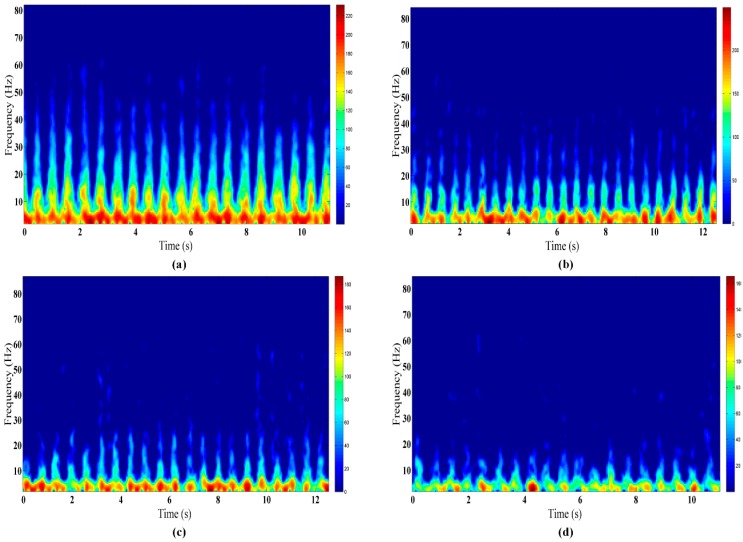
Spectrograms of the piaffe activity at different positions: (**a**) 3 m, (**b**) 4 m, (**c**) 5 m, and (**d**) 6 m.

To classify different human activities at changing positions, the current conventional method first acquires the overall SVM through training based on all data at all positions. Then, the classification of data at any position is realized based on the overall SVM. However, taking piaffe as an example (Figure 7), with the increase of the through-wall detection range, the high-frequency micro-Doppler signals which are clearly distinguishable in finer-grained human activities will gradually attenuate. This will easily cause the misjudgment of different activities at different range positions. Therefore, based on the pre-existing mature technology for human through-wall range positioning via UWB radar, this paper proposes a new classification method by OS-SVM based on prior human position information. 

**Figure 8:**
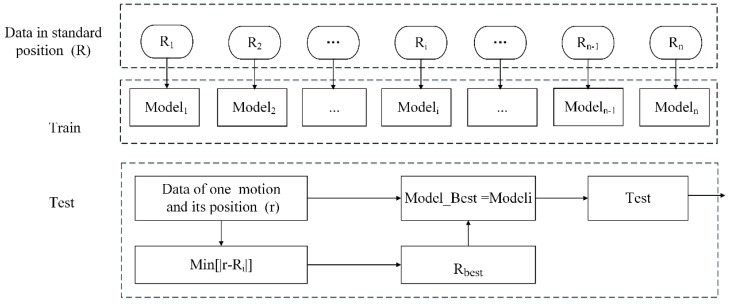
Operation diagram of OS-SVM based on prior human position information.

A schematic diagram of the new classification method is shown in Figure 8. In the training stage, the data obtained at different standard range positions $R=\left[R_{1},R_{2},\ldots ,R_{i},\ldots R_{n-1},R_{n}\right]$ are used to establish the corresponding pattern recognition models *Model* = [*Model*_1_, *Model*_2_,…, *Model_i_*,…,*Model_n_*_−1_, *Model_n_*]. In the testing stage, according to the prior human position information ***r*** obtained from the SFCW radar, the best model—$Model_{Best}$—is determined automatically based on the principle of proximity. Then, the classification of the motion feature data at this position will be conducted based on$\;Model_{Best}$.

In this research, the ***R*** values at the standard position are as follows: $R=\left[R_{1},R_{2},R_{3},R_{4}]=[3,4,5,6\right]$ (unit: m), and the pattern recognition models are *Model* = [*Model*_1_, *Model*_2_, *Model*_3_, *Model*_4_] During testing, for example, if the prior human position information ***r*** is equal to 4.6, $R_{best}=R_{3}$ is obtained according to the$\text{min}\left(\left|R_{i}-r\right|\right)$ principle, and the optimal model $Model_{Best}=Model_{3}$. Then, the human activity data at position ***r*** will be classified based on $Model_{3}$.

## 4. Experimental Results and Discussion

This section presents the verification and discussion of the effectiveness and superiority of the through-wall detection and classification technology for finer-grained human activities, based on a classification experiment of six finer-grained human activities at a fixed through-wall position and a second experiment of five finer-grained human activities at variable positions.

### 4.1. Pattern Recognition Training Feature Set Generation

When using the SVM algorithm to conduct data training and classification, the eight features extracted from the CRATFR spectrograms are taken as the eigenvalue group. As a special case in the changing-position mode, an experiment in the fixed-position is used to verify the effectiveness of the new CRATFR algorithm and the eigenvalues obtained in this paper. During data acquisition in the fixed position, the subject faces the radar behind the wall at a distance of 3 m (±0.3 m) from the radar. The data set is obtained by extracting the features of the measured data acquired for eight subjects performing six activities. Each subject performed each activity twelve repeats for a period of 8 s per repetition. Thus, the total number of the features set is 576.

The mean values of the eight features of the six activities are shown in Table 1. Clearly, they are quite different, and therefore, they may be reliably and reasonably applied in the recognition and classification system.

For the classification experiment of the different activities at varying through-wall positions, the data from five activities (excluding breathing) at positions of 4, 5, and 6 m (±0.3 m) were also acquired, in addition to the existing 3 m data described above. During data collection, every activity at each position was collected twelve repeats. Ultimately, 1920 feature sets were obtained.

### 4.2. Fixed-Position Human Activity Classification 

In this test, we used 3/4 of the data obtained at fixed positions as the training data set and the remaining was used to generate the validation data set.

**Figure 9:**
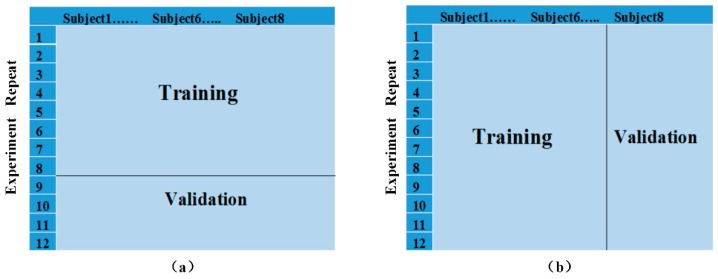
Schematic diagrams of the two selection scenarios. (**a**) The first scenario; (**b**) The second scenario.

When using LibSVM, two scenarios can occur during the selection of the training and validation data (Figure 9). The first scenario selects eight repeats from each subject as the training data, and the remaining four repeats as the validation data. The second scenario selects all the repeats of 6 subjects as the training data, and uses the data from the remaining two subjects as the validation data. The latter would more closely reflect a practical application, and thus, is more realistic and practical because it classifies the activities of unknown people based on the information of known people. 

The final judgment of accuracy and the corresponding optimal parameters *c* and *g* are obtained through the comprehensive averages of four-fold cross-validations. The data was divided into 4 quarters for training and testing purposes and the different errors relates to the error obtained when validating on each quarter of the data while training on the remainder.

To facilitate the graphic representation of the classification results, the six activities (piaffe, picking up an object, waving, jumping, standing with random micro-shakes, and breathing while sitting) are respectively numbered 1–6.

**Figure 10:**
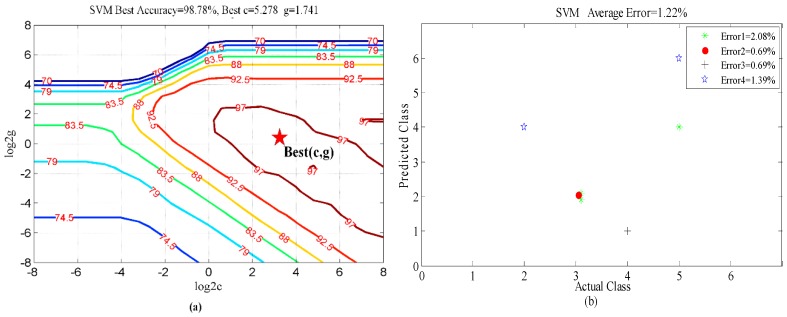
Classification results from four-fold cross-validation for the first scenario. (**a**) Parameter selection results; (**b**) Classification errors distribution of the four-fold cross-validation (the horizontal axis represents the actual activity class represented as the numbers 1–6, the vertical axis represents the predicted activity class represented as the numbers 1–6).

**Figure 11:**
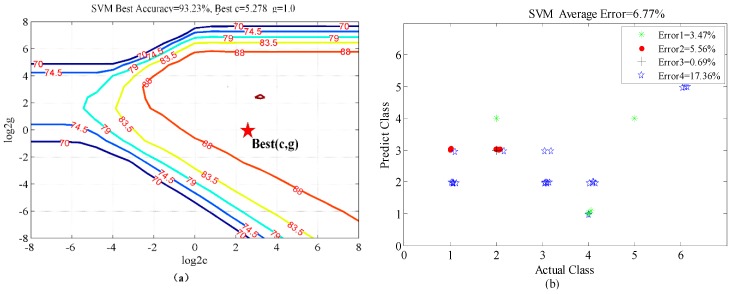
Classification results from four-fold cross-validation for the second scenario (**a**): The parameter selection results. (**b**) Classification errors distribution of the four-fold cross-validation.

According to the parameter selection results from the four-fold cross-validations shown in Figure 10a and Figure 11a, the best average classification accuracy of the six activities for the first scenario is 98.78%, with the corresponding best parameters selections *c* = 5.278 and *g* = 1.741. The best accuracy is 93.23% for the second scenario, with the best parameters selections *c* = 5.278 and *g* = 1.0. The first scenario has a slightly smaller validation error than the second one. It is probable that the target source of the testing data is the same as that of the training data in the first scenario, so that they share a certain similarity. The classification errors distributions of the four-fold cross-validations for the two scenarios are shown in Figure 10b and Figure 11b. The first scenario is more stable than the second one. For the more realistic case, *i.e.*, the second scenario, activities (2) (picking up an object) and (3) (waving) were inclined to be confused. This is probably because the Doppler features of picking up an object are similar to those of waving. Finally, the breathing while sitting activity (6) has the smallest classification error in both scenarios, because the Doppler signature of breathing is so small that it is clearly distinct from the other motions.

### 4.3. Changing-Position Human Activity Classification

In the classification tests of human activities with changing positions, to meet the requirements for practical application, we only focus on the second scenario proposed in this paper. Further, because of the sharp signal attenuation that occurs with the increasing through-wall detection range, it is difficult for the weak signal due to breathing while sitting to show clear and distinguishable features in the time-frequency spectrum. Thus, the other five activities were selected for the testing.

During the experiment, the reference method (*i.e.*, the conventional overall SVM classification method) was applied to classify all the data at all positions for the eight subjects. The data from six subjects at all positions were used as the training data to obtain the overall model. When testing, the overall model was used for classifying the data of the other two individuals at any position. However, the proposed OS-SVM method for the experimental group was able to achieve more efficient classification of four groups of data at positions of 3, 4, 5, and 6 m (±0.3 m) because *Model_i_* at each *R_i_* results from the training data of six persons at the corresponding positions, and the data from the remaining two subjects refer to validation data. Ultimately, the final average classification accuracy was obtained from the four-fold cross-validation. 

**Figure 12:**
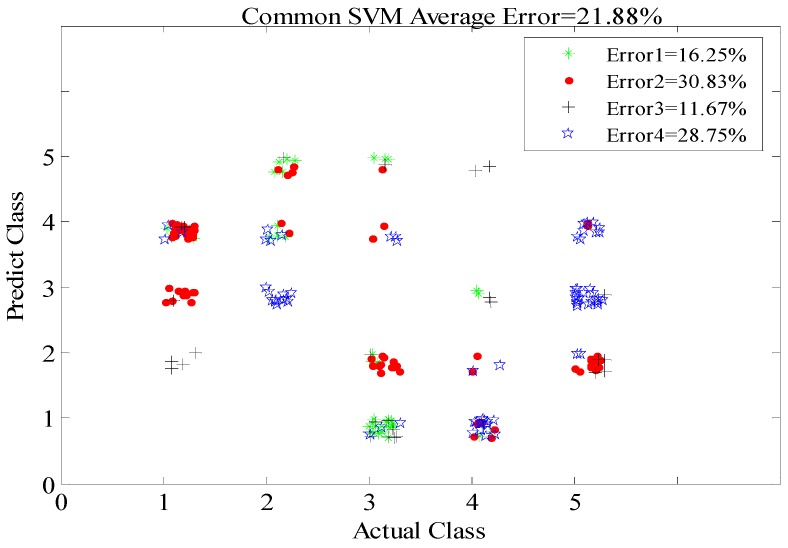
Four-fold cross-validation error distribution of the reference method at changing positions.

**Figure 13:**
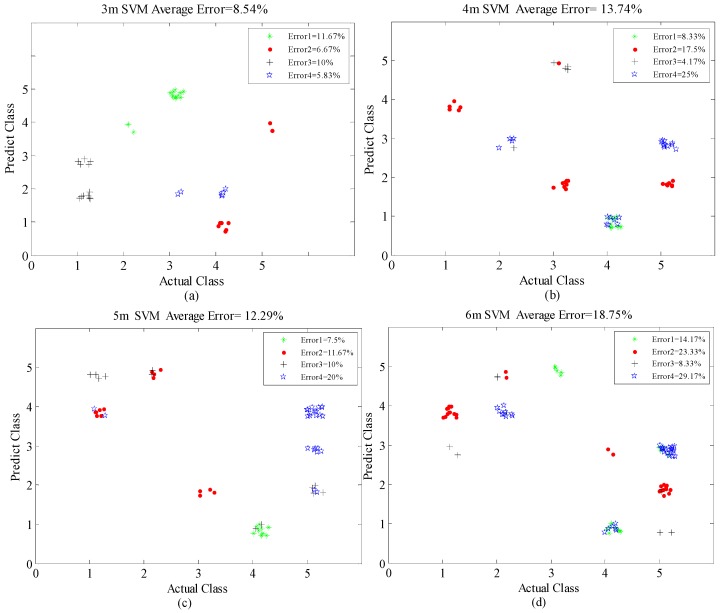
Four-fold cross-validation error distributions of proposed method at different positions: (**a**) 3 m, (**b**) 4 m, (**c**) 5 m, and (**d**) 6 m.

As shown in Figure 12, for the classification of human activities at changing positions within 6 m, the reference method produced a higher average rate of classification error, 21.88%, whereas the method proposed in this paper afforded excellent performance, with an average error of 13.33% (the mean value of 8.54%, 13.74%, 12.29%, and 18.75% for the four distances), as shown in Figure 13. In other words, the classification accuracies of the reference and proposed methods are 78.12% and 86.67%, respectively. This is because the training model of the OS-SVM is more similar to the eigenvalues of the actual motion signals in the corresponding positions than those in the reference method. Meanwhile, with the increase of through-wall detection range, the classification error rate increases gradually, from 8.54% at 3 m, 13.37% at 4 m, 12.29% at 5 m, and 18.75% at 6 m. This trend occurs because the increase of the through-wall range inevitably weakens the Doppler high-frequency features and reduces these feature differences among the different activities, thus increasing the misjudgment rate.

Furthermore, through the overall observation of Figure 13a–d, the probability of classification error caused by confusion between the (1) piaffe and (4) jumping motions is the highest. We think that this is because the high and low frequency features of the two activities are relatively strong and similar. Further, according to the results obtained at 5 and 6 m, we found that activity (5), standing with random micro-shakes, is easily misjudged as (2), picking up an object, or (3), waving, shown in Figure 13c,d. This shows that finer-grained human activities with fewer and weaker high-frequency micro-Doppler features are harder to classify when the through-wall detection range increases.

## 5. Conclusions

In this paper, we first selected SFCW UWB radar to conduct the through-wall detection of six common finer-grained human activities according to actual detection environments that would be encountered in applications such as anti-terrorism activities and security. We then introduced a comprehensive range-accumulation time-frequency transform (CRATFR) method based on an inverse weight coefficient according to the characteristics of the finer-grained human activity signals detected by the SFCW radar. This method can make full use of the time-frequency information of different range bins of the UWB radar signal, so as to enhance the micro-Doppler time-frequency spectral features of the finer-grained human activities. The results indicate that different activities show obvious feature differences in their time-frequency spectra.

After the time-frequency analysis of human activities, we classified the different motions by extracting eight effective eigenvalues from the spectra resulting from the CRATFR analysis and utilizing SVM. At a position 3 m behind a wall, the data classification accuracies for six activities of eight persons were 98.78% and 93.23%, respectively, for the two scenarios defined in this paper. Furthermore, to correct the deficiency wherein conventional SVM will easily misjudge activities at different through-wall ranges, this paper proposed a novel classification method, the optimal self-adaption support vector machine (OS-SVM), based on prior human position information. Accordingly, the model included in the OS-SVM better captures the time-frequency features of actual motion signals in the corresponding positions. At the same time, it avoids the misjudgment that easily occurs because of the similarity of the features of different activities in different positions which is caused by the weakening of the Doppler high-frequency signals. Experimentally, within the 6 m range, the average classification accuracy of five activities data of eight subjects is 86.67%, while the recognition accuracy of current common method is only 78.12%. Thus, this research is of great significance for the through-wall detection and classification of finer-grained human activities. With further research, it should provide important information for practical applications such as anti-terrorism activities.

## Figures and Tables

**Table 1:** Mean values of the eight selected features.

Activity	Feature (1)	Feature (2)	Feature (3)	Feature (4)	Feature (5)	Feature (6)	Feature (7)	Feature (8)
(1)	72.15	48.96	1.10	1.77	66.32	67.84	4.94	18.25
(2)	38.63	24.13	2.64	2.79	72.28	15.14	1.88	9.33
(3)	32.75	21.80	1.92	1.54	45.50	18.83	1.18	8.59
(4)	85.84	62.54	2.60	1.95	67.73	45.85	4.40	23.51
(5)	18.68	6.52	3.32	3.02	76.23	7.82	0.65	2.71
(6)	7.60	0	3.47	2.88	17.76	3.39	0	2.58
